# Study on Main Drugs and Drug Combinations of Patient-Controlled Analgesia Based on Text Mining

**DOI:** 10.1155/2020/8517652

**Published:** 2020-05-23

**Authors:** Xing Jin, Ying Wu

**Affiliations:** ^1^Department of Anesthesiology, Shanxi Cancer Hospital, Taiyuan 030013, China; ^2^School of Humanities and Social Sciences, Shanxi Medical University, Taiyuan 030001, China

## Abstract

In recent years, with the continuous understanding of pain knowledge and the continuous improvement of quality of life requirements, patient-controlled analgesia (PCA) has been widely used in a variety of pain patients. In this study, text mining technology was used to analyze relevant literature, try to find out the main drugs of PCA, classify the drugs, and dig out the important drug combination rules. PCA studies were retrieved from PubMed database in recent 10 years, and the bibliographic information of the literatures was taken as mining sample. First, the names of the drugs in the sample were identified by MetaMap package; then, Bicomb software was used to extract high-frequency drugs for the word frequency analysis and to construct a drug-sentence matrix. Finally, “hclust” package and “arules” package of R were used for the cluster analysis and association analysis of drugs. 39 main PCA drugs were screened out. Morphine, dexmedetomidine, and fentanyl were the top three drugs. Through cluster analysis, these drugs were divided into two clusters, one containing 26 common drugs and the other containing 13 core drugs. The association analysis of these drugs was carried out, and 22 frequent itemsets and 6 association rules were obtained. The maximum frequent 1-itemset was {Morphine} and the maximum frequent 2-itemset was {Morphine, Ropivacaine}. The research results have certain guidance and reference value for clinicians and researchers. In addition, it provides a way to study the relationship between drugs from the perspective of text mining.

## 1. Introduction

Pain is an unpleasant feeling and emotional sensation associated with existing or potential tissue damage, and it is listed as the fifth vital sign after temperature, pulse, respiration, and blood pressure [1]. In recent years, with the continuous understanding of pain knowledge and the continuous improvement of quality of life requirements, patient-controlled analgesia (PCA) has been widely used in a variety of pain patients. Therapeutic approaches include intravenous PCA (PCIA), epidural PCA (PCEA), subcutaneous PCA (PCSA), and peripheral nerve PCA (PCNA) [2, 3]. The common drugs used in PCA are opioid analgesics, low-concentration local anesthetics, and nonopioid analgesics, as well as some sedatives and antiemetic drugs. Opioids are the most commonly used analgesics in clinical practice [4]. These drugs have side effects such as nausea, vomiting, tolerance, and gastrointestinal dysfunction while treating pain. Respiratory inhibition is the main reason that limits the safety of opioids [5].

Multimodal analgesia (MMA) is the most effective analgesic strategy to reduce adverse reactions caused by a single drug or method, which combines various analgesic drugs and methods with different mechanisms to exert the best analgesic effect [6, 7]. In the treatment of PCA, more and more patients use MMA, and the types of drugs used and the ways of administration are more and more diversified. For example, adding ketamine to morphine/ hydromorphone PCA provides a small improvement in postoperative analgesia while reducing opioid requirements. Adjunctive ketamine also reduces postoperative nausea and vomiting without a detected increase in other adverse effects [8]. Adjunctive parecoxib during morphine combined with ropivacaine epidural PCA (PCEA) following abdominal hysterectomy is safe and efficacious in reducing pain [9]. The combination of dexmedetomidine and sufentanil for intravenous PCA (PCIA) after abdominal operation could reduce sufentanil consumption, decrease visual analogue scale (VAS) scores, lower the rate of nausea and vomiting, and improve patient satisfaction [10]. Faced with a large number of drugs and complex drug combination formulas, how to screen the main drugs in PCA and discover the combination rules of drugs and obtain valuable information from them becomes particularly important.

While a large number of experimental data are growing at an extraordinary rate, medical literatures are also growing at an explosive rate. A large number of literatures not only bring opportunities to obtain relevant information, but also contain many important links and rules. Therefore, they have also become data mining samples, resulting in text mining [11].

In this study, the text mining technology was used to analyze PCA-related literatures in the PubMed database of the National Library of Medicine of the United States, so as to try to find out the main drugs of PCA, classify the drugs, and dig out the important drug combination rules.

## 2. Materials and Methods

### 2.1. Data Collection

From PubMed database (http://www.ncbi.nlm.nih.gov/PubMed), we retrieved 735 relevant literatures using the terms “analgesia, patient-controlled [MeSH Major Topic],” with no restriction on the language; the retrieval time span was 10 years and ended at 2019/06/08. The full bibliographic information (including title and abstract) of all documents was saved in the TXT format and used as the sample.

### 2.2. Drug Identification

The drug names in the samples were identified by the MetaMap program [12] of National Medical Library (NLM). MetaMap is a natural language processing program developed by NLM. It can match free words in the TXT format text to concepts in the Metathesaurus of the Unified Medical Language System (UMLS). It can compare words in titles and abstracts with concepts in the Metathesaurus and find matches through certain algorithms. A semantic type is assigned to each word. UMLS divided concepts into 127 semantic types [13]. In this study, “Pharmacologic Substance” was used as the semantic type to identify drug names in the text sentence by sentence, and the identified drug names were automatically tagged with a semantic type.

### 2.3. Extracting Identified Drugs and Constructing Drug-Sentence Matrix

The word frequency analysis is an important means of text mining, which is a statistical analysis of the occurrence of important words in the text. Bicomb software [14] was used to extract the identified drugs, standardize the name of drugs, and delete the names of drugs (such as medicine and opioid agonist), which are too broad or unclear. The frequency of drug occurrence in the sample was counted, and the drugs were arranged in descending order. Then, according to Pao's effective word frequency formula [15] *T* = [1 + (1 + 8*I*_1_)^1/2^]/2 (*T* is the dividing frequency of high-frequency words and low-frequency words and *I*_1_ is the number of words appearing once) to judge and screen drugs to find high-frequency drugs.

In the process of identifying drug names, MetaMap first divided the text into sentences and then added semantic types to each drug names. Therefore, the Bicomb software [16] was used to construct the drug-sentence matrix based on sentence. If a drug appeared in the sentence, it was marked as “1;” otherwise, it was marked as “0.” If two or more drugs co-occurred in the same sentence, it was considered that there was a combination relationship between them.

### 2.4. Cluster Analysis and Association Analysis Based on R

There are many methods of text mining, among which clustering refers to classifying these data into different categories according to the similarity between the original data. System clustering is a common clustering method, which mainly designs the algorithm from the perspective of distance and connectivity. The idea of “bottom-up” is adopted in system clustering. First, each sample is regarded as a different small cluster, and the nearest small clusters are combined by repetition until all the samples belong to the same cluster. The results are generally deterministic and hierarchical. System clustering has the advantages of easy definition of distance and rule similarity, no need to set the number of clusters beforehand, and can find the hierarchical relationship of the cluster. However, if there are too many observations, the calculation will be too complex, the clustering results will form a chain, and it is difficult to analyze the hierarchical relationship. In this study, we used the “hclust” package [17] of R to cluster the generated discourse matrices and gradually aggregate the close drugs into a number of conceptually independent classes. Through the study and interpretation of these classes, the current situation and rules of combination drug are analyzed and predicted.

The association analysis is to describe and analyze whether there are symbiotic phenomena in existing data, mainly reflecting the relevance between things, that is, the possibility of some events happening together or the hidden rules of mutual relations between events, so as to complete the speculation of the known things on the unknown. The Apriori algorithm is a classical association rule algorithm and the core of all association mining algorithms. It has the advantages being simple and easy to understand and having low data requirements. However, because the algorithm needs to call data set repeatedly, it will lead to a long time and low efficiency when the data set is too large. The association analysis applied the “arules” package [18] of R, and used the Apriori to conduct data association relation mining. The process consisted of two stages. First, all frequent itemsets were found from the drug-sentence matrix. Association rules were then generated from these frequent itemsets. Through the interpretation of association rules, the tightest combinations of drugs were found.

## 3. Results

### 3.1. Results of High Frequency Drugs and Drug-Sentence Matrix

Through the extraction of drug names from the bibliographic information, 83 PCA-related drugs were obtained, including 19 drugs with a frequency of 1. According to Pao's effective word frequency formula, 39 high-frequency drugs with a lowest frequency 7 were determined, and the cumulative word frequency contribution rate was 93.10%. Morphine, dexmedetomidine, and fentanyl were the top three drugs (Table 1).

The drug-sentence matrix of 39 high-frequency drugs was constructed by Bicomb software, and 593 sentences with co-occurrence relationship were obtained. Part of the matrix is shown in Table 2.

### 3.2. Results of System Clustering

**Figure 1:**
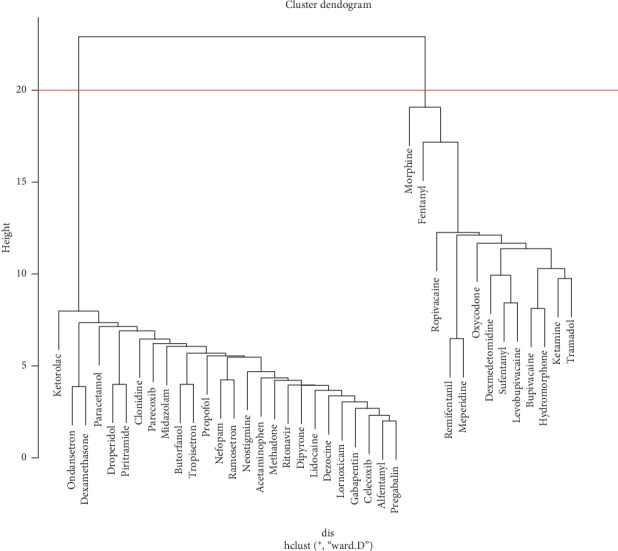
Cluster dendrogram of 39 high-frequency drugs related to PCA.

The clustering process was to integrate 39 drugs from small clusters to larger ones according to the distance, and the similarity within the cluster decreases gradually. The drugs in the smallest cluster can often be combined with drugs directly. In addition, we can make valuable discoveries by analyzing the nature or type of different clusters of drugs. The results of clustering are shown in Figure 1. According to the distance at the red line, the 39 drugs were generally divided into two big clusters.

The first cluster contained 26 drugs, with propofol appearing 78 times at the highest frequency and celecoxib appearing 7 times at the lowest frequency. Ketorolac is the most widely used combination drug, which was related to eight drugs, and litonavir, methadone, and pregabalin were the least, which were related with only one drug.

The second cluster contained 13 drugs, with morphine appearing 759 times at the highest frequency and meperidine appearing 57 times at the lowest frequency. Morphine was the most abundant combination drug, which was related with 26 drugs, and meperidine was related with 6 drugs at least.

### 3.3. Results of Frequent Itemsets and Association Rules

The Apriori algorithm involves three important parameters, support, confidence, and lift. Support measures the universality of the application of association rules, and the higher the degree of support, the more common the rule is adopted; confidence reflects the accuracy of association rules, and the higher the degree of confidence, the greater the opportunity of the latter item under the condition of the existence of the preceding item of the rule; lift reflects the practicability of the association rules, only the lift with a degree greater than 1 are useful. In order to obtain a certain number of the association analysis results, we set the support to 0.05 and the confidence to 0.7.

**Figure 2:**
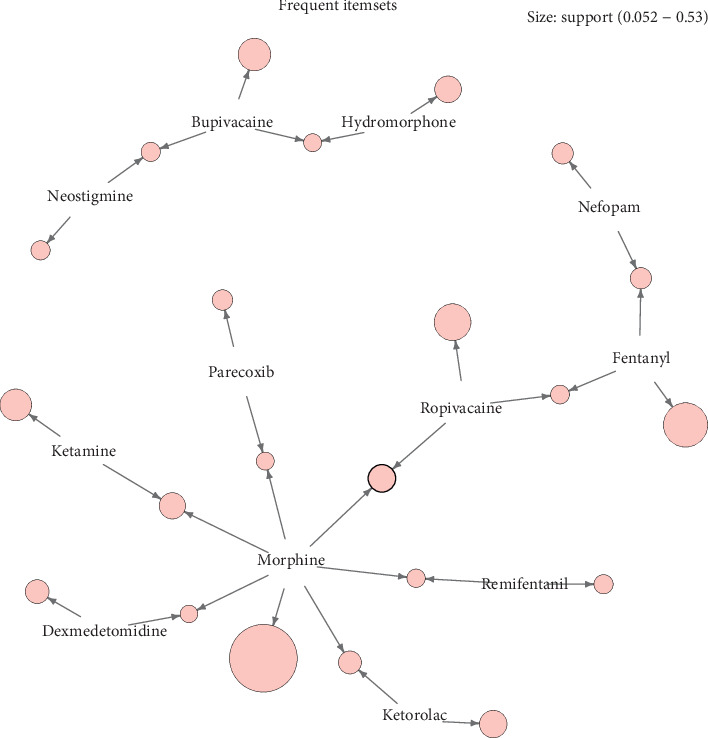
The visualization map of 22 frequent itemsets.

After operation, we got 22 frequent itemsets. Among them, there were 12 frequent 1-itemsets and 10 frequent 2-itemsets. As shown in Figure 2, the red circle represented support, with a larger circle indicating greater support. Each item pointed to support through a directed arrow, indicating that the relevant items constitute an itemset. In this case, support varied from 0.052 to 0.53. The maximum frequent 1-itemset is {Morphine} with support of 0.53; the maximum frequent 2-itemset is {Morphine, Ropivacaine}, with support of 0.15.

**Figure 3:**
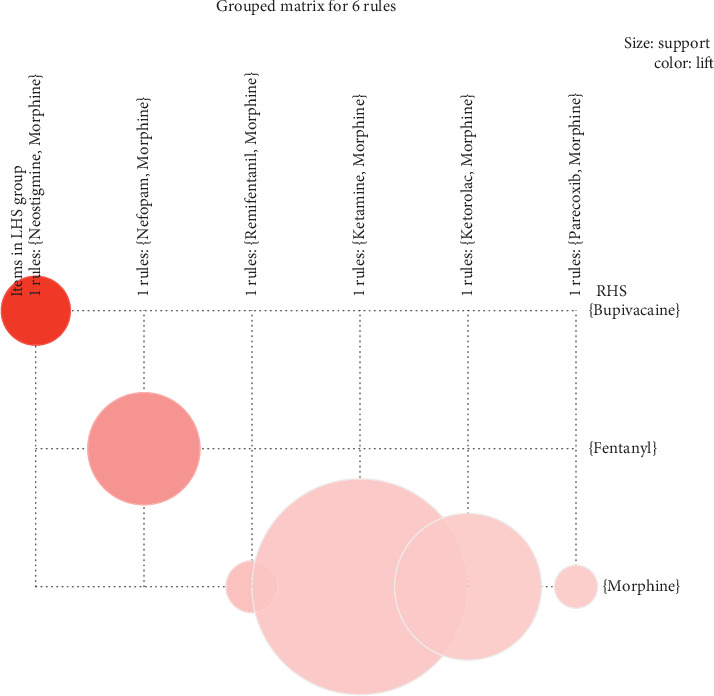
The visualization map of 6 association rules.

In the analysis of association rules, we obtained six valuable association rules with lift greater than 1. As shown in Figure 3, a larger circle indicated greater support; a darker color indicated greater lift. Among them, {Ketamine} ≥ {Morphine} was an association rule with the maximum support of 0.14. The association rule with the maximum lift was {Neostigmine} ≥ {Bupivacaine}, with the lift of 5.12.

## 4. Discussion

First, in the word frequency analysis, 39 high-frequency drugs of PCA were obtained. Morphine, dexmedetomidine, and fentanyl were the top three drugs; these drugs are the main drugs for PCA. As a classic opioid receptor agonist, morphine has a definite analgesic effect and a long duration, which is a common drug for PCA. The application of morphine in PCA is flexible and extensive. It can be used alone or combined with other analgesic drugs, and there are many ways of administration, such as intravenous, epidural, and subcutaneous. Fentanyl is a newer opioid receptor agonist, which has the advantages of rapid onset and stable cardiovascular system function. Some studies have shown that compared with morphine, fentanyl used in PCA can reduce adverse reactions and shorten the length of hospital stay [19]. Dexmedetomidine, as an alpha 2-adrenoceptor agonist, can be combined with a variety of analgesics to enhance the analgesic effect. When dexmedetomidine is combined with opioids, the dosage of opioids can be reduced, and the incidence of adverse reactions can be reduced on the premise of maintaining the analgesic effect [20]. Through the drug-sentence matrix, we can find that 39 drugs all have co-occurrence relationship with other drugs, which indicates that drug combinations are common in PCA.

Second, in the systematic cluster analysis, two clusters of drugs were obtained. The first cluster was common drugs, which were rarely combined with other drugs and mainly used as adjuvant drugs. Most of them were nonsteroidal anti-inflammatory drugs, while a small amount of antiemetics and local anesthetics were also available. Generally speaking, they were difficult to be used alone for PCA, so they should be combined with other powerful analgesics. The second cluster was core drugs, which were widely combined with other drugs and were the core analgesics of PCA. Most of them were opioid powerful analgesics, and there were also long-term local anesthetics, which were widely used, such as morphine, fentanyl, and ropivacaine; they can be used as a single drug for PCA [21–23]. Among these drugs, dexmedetomidine, ranked second in frequency, is not an opioid and has both sedative and analgesic effects. It had a combination relationship with many drugs and played an important role in PCA combination drugs.

Finally, the Apriori algorithm is applied to analyze the drug-sentence matrix, and 22 frequent itemsets and 6 association rules were obtained. Drugs in frequent itemsets were more closely related to the combination of other drugs. The Apriori algorithm starts from the bottom (1- itemset), uses iterative method to find the supersets of the lower layer, and finds frequent itemsets in the supersets according to the support. Therefore, the maximum frequent 1-itemset was {Morphine} and the maximum frequent 2-itemset was {Morphine, Ropivacaine} suggested that morphine was most likely to appear in all PCA formulas; in multi-drug combination formulas, “Morphine + Ropivacaine” was more likely to appear. Ropivacaine is a relatively safe local anaesthetic with a better safety margin and separation of sensory and motor effects. Morphine combined with ropivacaine can be used in many kinds of analgesia, such as PCEA, caudal analgesia, and continuous intrathecal analgesia [24–26]. The combination of the two drugs can reduce the dosage of morphine on the premise of maintaining or enhancing the analgesic effect, so as to reduce the side effects of opioids. From the results of association rules, six closely related drug combinations were obtained, which involve two important measures of support and lift. Support is a measure of the validity of rules. The greater the support is, the greater the probability that the preceding item and the latter item will appear together. The association rule with the maximum support was {Ketamine} ≥ {Morphine}, which indicated that if ketamine was used in the PCA formulation, and morphine would be more likely to be used in combination. Lift is a measure of the practicability of rules. The greater the lift is, the greater the influence of the preceding item on the latter item. {Neostigmine} ≥ {Bupivacaine} was the association rule with the maximum lift, which indicated that if neostigmine was used in the formulation, it was more reasonable to use bupivacaine in combination.

There were some limitations in this study. In order to match the MetaMap program better, we only chose PubMed database as the only source of literature data; we will add more medical professional database in the future research. In addition, due to the co-occurrence method used in the extraction of drugs, there was a lack of negative detection. Even if it was explicitly stated in the same phrase that there was no relationship between certain drugs, they were still be considered to have co-occurrence relationship, impacting the results of the study.

## 5. Conclusions

In the treatment of pain with PCA, people advocate MMA. The combination of different pharmacological analgesics can reduce the side effects of opioids and maintain an adequate analgesic level. In this study, text mining technology was used to mine recent literatures on PCA, identify the main drugs, and analyze the core drugs in combination drugs and the association between them. The results of this study have certain guidance and reference value for clinicians and researchers. In addition, it provides a way to study the relationship between drugs from the perspective of text mining.

## Figures and Tables

**Table 1:** 39 high-frequency drugs related to PCA.

Rank	Drug name	Frequency
1	Morphine	759
2	Dexmedetomidine	398
3	Fentanyl	360
4	Remifentanil	333
5	Ropivacaine	162
6	Sufentanil	129
7	Oxycodone	121
8	Ketamine	107
9	Bupivacaine	105
10	Tramadol	99
11	Propofol	78
12	Hydromorphone	73
13	Levobupivacaine	70
14	Meperidine	57
15	Paracetamol	47
16	Parecoxib	46
17	Ketorolac	46
18	Droperidol	36
19	Clonidine	36
20	Alfentanil	33
21	Ondansetron	29
22	Acetaminophen	28
23	Neostigmine	27
24	Nefopam	27
25	Midazolam	25
26	Dexamethasone	21
27	Lidocaine	19
28	Dezocine	19
29	Piritramide	19
30	Ramosetron	19
31	Butorfanol	18
32	Tropisetron	15
33	Pregabalin	14
34	Methadone	14
35	Gabapentin	14
36	Lornoxicam	11
37	Dipyrone	9
38	Ritonavir	8
39	Celecoxib	7

**Table 2:** Part of the drug-sentence matrix.

Drug name	1	2	3	4	5	6	7	8
Morphine	0	1	1	1	1	0	0	0
Dexmedetomidine	0	0	0	0	0	0	0	0
Fentanyl	0	0	0	0	0	1	0	0
Remifentanil	0	0	0	0	0	0	0	0
Ropivacaine	1	1	0	1	0	0	1	0
Sufentanil	0	0	0	0	0	0	0	1
Oxycodone	0	0	0	0	0	0	0	0
Ketamine	0	1	0	0	0	1	0	0
Bupivacaine	0	0	0	0	0	0	0	0

## Data Availability

The data used to support the findings of this study are available from PubMed database.
